# Admission hypomagnesemia as a mortality predictor in medical critically ill patients

**DOI:** 10.1186/cc13621

**Published:** 2014-03-17

**Authors:** A Permata Sari, C Pitoyo, D Aditianingsih, C Rumende

**Affiliations:** 1 Medical Faculty Indonesia University, Jakarta Pusat, Indonesia

## Introduction

Magnesium is the second most abundant intracellular cation and serves as a cofactor in more than 300 enzymatic reactions. Hypomagnesemia is a common electrolyte imbalance in critically ill patients; yet it is frequently overlooked. Previous studies highlighted the relationship between hypomagnesemia and mortality in these patients [1,2]. This study was carried out on patients admitted to the critical care unit and seeks to find admission hypomagnesemia's role as a 28-day mortality predictor in medical critically ill patients.

## Methods

This is a cohort prospective study with prognostic research recruiting 150 critically ill patients in a major tertiary hospital. Blood samples were collected for estimation of serum total magnesium on admission, and then the patients were followed over 28 days.

## Results

This study was conducted consecutively from April to July 2013. Most subjects were male (62%) with mean age 52.6 ± 15.93 years. The occurrence of sepsis (33.3%) and cardiac disturbances (24.7%) were the most common problems among patients. The mean MSOFA score for the hypomagnesemics was higher than the normal group (4.91 ± 4.19 vs. 3.77 ± 3.52). Subgroup analysis in the MSOFA 0 to 7 group had significant *P *= 0.015 (chi-square) and crude RR = 2.2 (95% CI = 1.19 to 4.06). From survival analysis, the survival mean of hypomagnesemia patients was lower than the normal group (19.4 vs. 22.8 days), and so was the survival percentage (52.7% vs. 74.7%).

## Conclusion

There was a high prevalence of hypomagnesemia in medical critically ill patients and it was associated with a high mortality rate.
